# Magnetoelectric Multiferroicity and Magnetic Anisotropy in Guanidinium Copper(II) Formate Crystal

**DOI:** 10.3390/ma14071730

**Published:** 2021-04-01

**Authors:** Pavla Šenjug, Jure Dragović, Filip Torić, Ivor Lončarić, Vito Despoja, Kristina Smokrović, Edi Topić, Ivica Đilović, Mirta Rubčić, Damir Pajić

**Affiliations:** 1 Department of Physics, Faculty of Science, University of Zagreb, Bijenička cesta 32, 10000 Zagreb, Croatia; psenjug@phy.hr (P.Š.); jure.dragovic@windowslive.com (J.D.); ftoric@phy.hr (F.T.); 2 Ruđer Bošković Institute, Bijenička cesta 54, 10000 Zagreb, Croatia; Ivor.Loncaric@irb.hr; 3 Institute of Physics, Bijenička cesta 46, 10000 Zagreb, Croatia; vdespoja@ifs.hr; 4 Department of Chemistry, Faculty of Science, University of Zagreb, Horvatovac 102a, 10000 Zagreb, Croatia; ksmokrovic@chem.pmf.hr (K.S.); etopic@chem.pmf.hr (E.T.); idilovic@chem.pmf.hr (I.Đ.); mirta@chem.pmf.hr (M.R.)

**Keywords:** multiferroics, metal-organic perovskites, magneto-electric effect, magnetic anisotropy, canted antiferromagnet

## Abstract

Hybrid metal-organic compounds as relatively new and prosperous magnetoelectric multiferroics provide opportunities to improve the polarization, magnetization and magneto-electric coupling at the same time, which usually have some limitations in the common type-I and type-II multiferroics. In this work we investigate the crystal of guanidinium copper (II) formate [C(NH${}_{2}$)${}_{3}$]Cu(HCOO)${}_{3}$ and give novel insights concerning the structure, magnetic, electric and magneto-electric behaviour of this interesting material. Detailed analysis of crystal structure at 100 K is given. Magnetization points to the copper (II) formate spin-chain phase that becomes ordered below 4.6 K into the canted antiferromagnetic (AFM) state, as a result of super-exchange interaction over different formate bridges. The performed ab-initio colinear density functional theory (DFT) calculations confirm the AFM-like ground state as a first approximation and explain the coupling of spin-chains into the AFM ordered lattice. In versatile measurements of magnetization of a crystal, including transverse component besides the longitudinal one, very large anisotropy is found that might originate from canting of the coordination octahedra around copper (II) in cooperation with the canted AFM order. With cooling down in zero fields the generation of spontaneous polarization is observed step-wise below 270 K and 210 K and the effect of magnetic field on its value is observed also in the paramagnetic phase. Measured polarization is somewhat smaller than the DFT value in the *c*-direction, possibly due to twin domains present in the crystal. The considerable magneto-electric coupling below the magnetic transition temperature is measured with different orientations of the crystal in magnetic field, giving altogether the new light onto the magneto-electric effect in this material.

## 1. Introduction

Multiferroics are materials with coexistence of more than one long-range order. Particularly interesting are magnetoelectric multiferroics, where a significant coupling between magnetic and ferroelectric orders is present, thereby allowing changes of magnetization with an electric field and changes of polarization with a magnetic field [1,2,3]. These materials are interesting because of the possibilities they offer in technological applications, such as in sensors, ferroelectric photovoltaics, spintronics and nanoelectronics [4,5,6,7]. In addition to technological applications, multiferroics are also important for fundamental research of the interactions between electron charge, spin, orbital degrees of freedom and crystal lattice [8].

It is very difficult to find magnetoelectric multiferroics with strong magnetoelectric coupling. The reason is mutually exclusive conditions for the existence of magnetic and electric order—magnetism requires partially filled *d* orbitals, while ferroelectricity usually requires a configuration of filled *d* orbitals [9]. According to the microscopic cause of ferroelectricity, multiferroics can be divided into two groups—type-I and type-II multiferroics. Type-I multiferroics are those materials in which the electric and magnetic order have different origins and appear almost independently of each other. Phase transition temperatures are different and spontaneous polarization is of large value (order of magnitude of 10–100 μC/cm${}^{2}$). The best known example of a type-I multiferroic is bismuth ferrite with phase transition temperatures 1100 K (ferroelectric) and 643 K (antiferromagnetic), and a polarization of 90 μC/cm${}^{2}$ [10] but with negligible magneto-electric coupling. Type-II multiferroics are the materials in which the origin of electric order is in magnetism. Usually, these are materials with a magnetic spiral order, and polarization occurs as a consequence of spin-orbit interactions. The coupling between the orders is large, but the polarization in type-II multiferroics is usually much smaller (of the order 10${}^{-2}$ μC/cm${}^{2}$) [2].

To avoid the problem of small coupling between the electric and magnetic orders in type-I multiferroics, and the problem of low magnetization and polarization in type-II multiferroics, composite multiferroics can be made where magnetic and ferroelectric materials are combined in the form of multilayered structures or self-organizing nanostructures [6]. Another way could be to use hybrid organic-inorganic materials. Most often, the inorganic part contains magnetic ions with a partially filled *d* orbital and is responsible for magnetism and magnetic order, while polarization and electric order occur as a consequence of the arrangement of organic blocks. By carefully selecting the organic groups, the desired physical properties can be obtained [11]. Hybrid organic-inorganic perovskites are one example of such materials. Perovskites are materials with a ABX_3_ formula, where A and B are cations of different sizes, and X anions form an octahedral coordination environment around the B cation. Corner sharing octahedra form a 3D network with the cavities in which A cations are located. A group of perovskites in which the A cation and/or X anion is replaced by organic cations and organic ligands, respectively, are called hybrid organic-inorganic perovskites. A large selection of organic groups of different structural and chemical properties provides the ability to adjust physical properties by simple chemical changes [12,13,14].

In already well known metal-formate perovskite, amine cations are located at the A sites and HCOO^−^ anions at the X sites [15]. Formate anions, due to their size, allow only weak magnetic interaction of neighboring magnetic ions. As a consequence, a long range magnetic order in such materials usually occurs only below 50 K. The *anti-anti* configuration of the formate anion causes the tilting of adjacent octahedra, which provides the possibility of non-centro-symmetric bridging and appearance of anti-symmetric super-exchange, that is, Dzyaloshinskii-Moriya interaction, which results in canted spins and weak ferromagnetism [12]. The ordering of organic cations at A positions leads to the structural phase transitions. At higher temperatures, usually there are several equivalent ways in which organic cations couple through hydrogen bonds to formate bridges. By cooling, they order by bonding in the same way and as a result of ordering, spontaneous polarization can occur. The first found example with such a structural transition in metal-organic perovskite is [DMA]M(HCOO)_3_], where DMA is a dimethylammonium ion (CH_3_)_2_NH_2_^+^, and M is a divalent metal ion [16,17].

Here, we study the metal-formate perovskite where the amine cation located at the A sites is guanidinium (Gua) cation, with formula C(NH${}_{2}$)${}_{3}^{+}$, having six hydrogen atoms with which it can form three pairs of hydrogen bonds with the oxygen atoms in formate bridges. Properties of several guanidinium metal formates, [Gua]M(HCOO)_3_ with divalent metal ions Mn${}^{2+}$, Fe${}^{2+}$, Co${}^{2+}$, Ni${}^{2+}$, Cu${}^{2+}$, and Zn${}^{2+}$ were investigated in [18]. They showed that compounds with magnetic ions Mn^2+^, Fe^2+^, Co^2+^, Ni^2+^ and Cu^2+^ have long-range spin canted antiferromagnetic order with transition temperatures of 8.8 K, 10.0 K, 14.2 K, 34.2 K and 4.6 K, respectively. In the compound [C(NH${}_{2}$)${}_{3}$]Cu(HCOO)${}_{3}$, abbreviated as GuaCuF, low-dimensional magnetism (AFM chains) is present at higher temperatures, which can be explained by the crystal structure in which the elongated octahedra (Jahn-Teller effect) are arranged to form Cu-formate-Cu chains with a smaller distance of copper ions than between copper ions from adjacent chains. Experimental and theoretical research has shown that GuaCuF has a ferroelectric order that occurs due to Jahn-Teller distortion of octahedra in a metal-formate network, which causes ferroelectric shifts of guanidinium cations through hydrogen bonds [19,20]. Magnetoelectric coupling was also observed [20].

In this work, we discuss the crystal structure of GuaCuF at 100 K. The performed ab-initio DFT calculation confirmed the nature of the magnetic ground state in agreement with the magnetization of powder and with the previous reports. The magnetization of a GuaCuF crystal was thoroughly measured, and very large anisotropy was found. The polarization was confirmed to be present even in the crystals with twin domains, somewhat lower than value obtained in DFT, and the effect of magnetic field on its value was observed even in the paramagnetic phase. The magnetoelectric coupling below the magnetic transition temperature was measured with different directions of electric field and orientations of the crystal in the magnetic field, from which the new light onto the appearance of magnetoelectric effect in this material is given.

## 2. Materials and Methods

### 2.1. Synthesis

Guanidinium copper (II) formate [C(NH${}_{2}$)${}_{3}$][Cu(HCOO)${}_{3}$] was synthesized by a slight modification of the previously reported procedure [18]. A fresh solution of formic acid (0.23 g, 6 mL water) was mixed with [C(NH${}_{2}$)${}_{3}$]${}_{2}$CO${}_{3}$ (0.38 g, 2.1 mmol). To the above solution, a solution of copper(II) nitrate (0.10 g Cu(NO${}_{3}$)${}_{2}\cdot$3 H${}_{2}$O dissolved in 2 mL of water) was added and the final mixture was allowed to evaporate slowly at room temperature. After a few days large blue prismatic crystals formed and were harvested by filtration. Yield: 77% based on Cu(NO${}_{3}$)${}_{2}\cdot 3$H${}_{2}$O. Elemental analysis calculated (%) for C${}_{4}$H${}_{9}$N${}_{3}$O${}_{6}$Cu: C 18.57, H 3.51, N 16.24; found: C 18.54, H 3.55, N 16.19.

**Figure A1:**
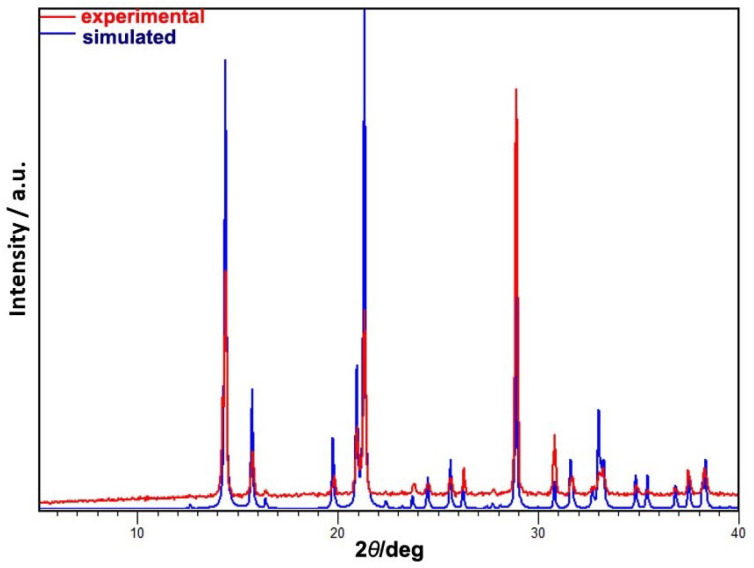
Comparison of the powder pattern for the synthesized material and the pattern simulated from the single crystal data (CSD Refcode: YUKVOQ) [18].

The purity of bulk material was confirmed via powder X-ray diffraction (PXRD) experiment performed on Panalytical Aeris diffractometer (Malvern Panalytical B. V., Almelo, The Netherlands) in Bragg-Brentano geometry (Figure A1).

### 2.2. Single-Crystal X-ray Diffraction

A crystal of appropriate diffraction quality was chosen and mounted on a glass needle. The data were collected via ω-scans at 100 K on an Oxford Xcalibur diffractometer (Oxford Diffraction Ltd, Abingdon, UK) having four-circle kappa geometry goniometer, Sapphire 3 detector and graphite-monochromated Mo Kα radiation (λ = 0.71073 Å). The data reduction and the analytical absorption correction were performed with the CrysAlis software package [21,22].The structure was solved by direct methods and refined against $F^{2}$ by the weighted full-matrix least-squares method by using programs SHELXS (Version 2013/1) [23] and SHELXL-2018/3 [24] operating under the WinGX system [25]. Selected crystallographic and refinement data for the title compound are summarized in Table A1. The structure was solved in $Pna2_{1}$ space group, while the Flack parameter refined to 0.45(2). The structure of the compound, previous to this report, was investigated *via* single-crystal X-ray diffraction in the temperature range 120–300 K (see [18,20,26]) and by neutron diffraction (on a deuterated analogue) in the temperature range 30–300 K (see [27,28,29,30,31]). The studies unveiled that the system does not suffer from symmetry related transitions in the low temperature region (30–300 K; see [27,28,29,30,31]). While the systematic absences allow the choice of the centrosymmetric Pnam (Pnma in standard setting) and non-centrosymmetric $Pna2_{1}$ space groups, based on the previous structural studies, extensive DFT calculations and the ferroelectric behaviour of the compound, the polar $Pna2_{1}$ space group was chosen. The value of the Flack parameter, in this particular case, was interpreted in the context of (racemic) twinning. Finally, it should be noted that the Pnan (Pnna in standard setting) is considered to be a spacegroup of the paraelectric phase for this compound. All non-hydrogen atoms were refined anisotropically. Hydrogen atoms bonded to carbon atoms were placed in geometrically idealized positions and refined using the riding model. Hydrogen atoms attached to nitrogen atoms of guanidinium cation were located in the difference Fourier maps at final steps of the refinement. Their coordinates were refined freely, but N-H distances were restrained to 0.88 Å. The structural analyses and geometrical calculations were done with PLATON [32,33], while the drawings were made with Mercury [34], POV-Ray [35] and Diamond [36]. Selected bond distances and angles are presented in Table A2, whereas hydrogen bond geometry is given in Table A3. CCDC 2058552 contains the supplementary crystallographic data for this paper. These data can be obtained free of charge via https://www.ccdc.cam.ac.uk/structures/, by emailing data_request@ccdc.cam.ac.uk, or by contacting The Cambridge Crystallographic Data Centre, 12 Union Road, Cambridge CB2 1EZ, UK; Fax: +44-1223-336033.

### 2.3. Density Functional Theory (DFT) Calculations

All DFT calculations were performed using a plane-wave code Quantum ESPRESSO [37,38] with GBRV (Garrity-Bennett-Rabe-Vanderbilt) pseudopotentials [39] and PBE (Perdew-Burke-Ernzerhof) exchange-correlation functional [40]. The energy cut-off for the plane wave basis set was set to 680 eV. Relaxation of ionic positions starting from experimental structure was performed until forces on all atoms were smaller than 0.01 eV Å^–1^ and change in energy of two consecutive steps was smaller than 0.5 meV. The Brillouin zone was sampled with a Monkhorst-Pack mesh with a density of k-points of at least 2.5 Å. Magnetic interaction parameters were calculated by fitting total energies of different spin configurations to the Heisenberg Hamiltonian. Polarization was calculated within so-called modern theory of polarization [41] as implemented in Quantum Espresso.

### 2.4. Magnetic Measurements

Magnetic properties were investigated on the polycrystalline powder sample and on the single crystals of guanidinium copper (II) formate using a MPMS 5 commercial superconducting quantum interferometer device (SQUID) magnetometer (Quantum Design, San Diego, CA, USA). MPMS 5 magnetometer enables the measurements of magnetization in the temperature range of 2–400 K, and in fields up to 55 kOe. For the powder sample, the ampule filled with powder was inserted into the measuring straw as a sample-holder, while the crystals were attached on a small piece of circular paper support so that the crystal is placed in the centre of the straw which is important for the measurement of transverse component of magnetization. The temperature dependence of magnetization *M*(*T*), was measured from 2 to 330 K, and higher temperatures were not used in order to avoid the grease melting and decentering or reorientation of the sample, as well as to stay far from the thermal instability of the sample which appears somewhat above 400 K. For several applied fields, *M*(*T*) was measured two times, first the sample was cooled in zero field and then measured in applied magnetic field while heating (the zero field cooled—ZFC curve), and the second time also while heating but after the sample was cooled down in the same applied field as the measuring one (the field cooled—FC curve). The field dependence of magnetization *M*(*H*) was measured at several stable temperatures in magnetic field up to 50 kOe. Besides the usually measured longitudinal magnetization in the direction of vertically applied magnetic field, the transverse component of magnetization which is in horizontal plane, that is, the component that is perpendicular to the applied field, was also measured. The horizontal sample rotator enabled the sample rotation in the horizontal plane and maximal magnetization during this rotation corresponds to the horizontal component of the magnetization vector. In such a way, the complete vector of magnetization is determined, consisting of the longitudinal (vertical) and transverse (somewhere in horizontal plane) components.

### 2.5. Magneto-Electric Measurements

Magneto-electric measurements were performed on the homemade modified sample-rod with wires going down to the sample within MPMS 5 magnetometer. The wires were made from the low thermal conductivity electric conductor in order to keep the lowest possible heat transmission and to ensure thermal stability of the sample-space down to 1.8 K.

For making the electric contacts to the crystal sample, the silver paste was used. The contacts were applied in such a way that the electric field goes parallel with the crystallographic *c*-axis. Namely, in this direction spontaneous polarization develops and can be measured most appropriately.

**Figure A2:**
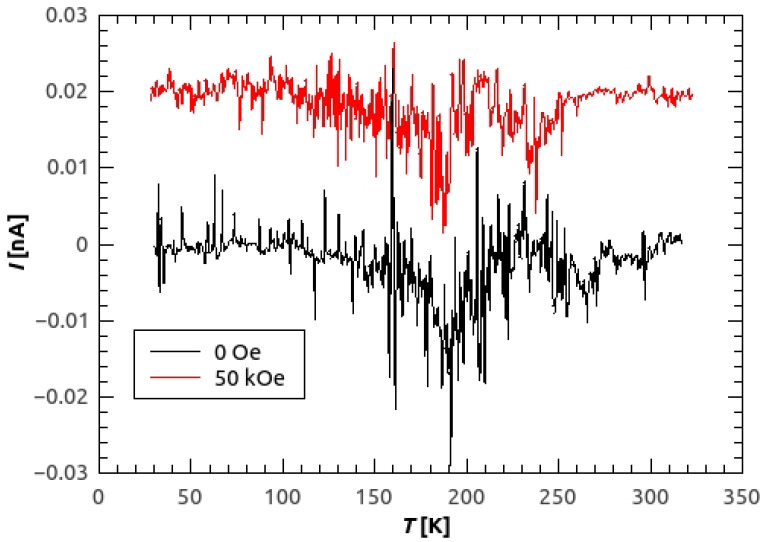
Pyroelectric current of [C(NH${}_{2}$)${}_{3}$]Cu(HCOO)${}_{3}$ in 0 Oe (black line) and in 50 kOe (red line). For the measurements in 50 kOe, the constant of 0.02 nA was added in order to separate the curves by translation.

The polarization was calculated from the measurements of the pyroelectric current by integrating it with time. Pyroelectric current flows due to the charges generated on the surface of crystal during the establishing of the electric polarization. Current was measured with Keithley 6517B electrometer (precise femtoampermeter) (Keithley, Cleveland, OH, USA) while cooling, in zero electric and magnetic field, from 330 K with constant rate of 2 K/min. The measurement was repeated for 50 kOe magnetic field applied in *a*-direction. We did not use the electric field for poling and measurement, in order to find out the spontaneous generation of polarization while cooling. On Figure A2 measured temperature dependence of pyroelectric current is shown for both 0 and 50 kOe magnetic field.

The magneto-electric effect was measured as an effect of electric field on the temperature dependence of magnetization. The electric field was applied using a high voltage source SRS-PS350 (Stanford Research Systems, Sunnyvale, CA, USA). Maximum used value of applied voltage was 250 V resulting in the field of around 2.5 kV/cm. Magnetization was measured two times in zero magnetic field, first the sample was cooled down in zero electric field and measured while heating, then above the magnetic order temperature the electric field was applied, and the sample cooled down to 2 K and measured again while heating.

### 2.6. Differential Scanning Calorimetry (DSC)

DSC measurement was performed on a single crystal of the title compound. The measurement was conducted on a TA DSC 25 instrument (TA Instruments Inc., New Castle, DE, USA) in a temperature range from 233 K to 323 K in a dynamic nitrogen atmosphere (50 mL/min) using TZero aluminium pans (40 μL). Heating and cooling rates were set at 2 K/min.

## 3. Results and Discussion

### 3.1. Molecular and Crystal Structure

At 100 K the title compound crystallizes in an orthorhombic system in a $Pna2_{1}$ space group. As established previously by neutron diffraction studies on a deuterated analogue (30–300 K) and by single-crystal X-ray diffraction in the temperature interval 120–300 K [20], the compound does not experience symmetry-related transitions in this low-temperature region [27,28,29,30,31]. It was recognized that in the 120–300 K temperature range all three cell axes expand with increasing temperature, although the crystallographic *b*-axis changes only slightly [20]. Additionally, it was shown that around 120 K unit cell *b*-axis exhibits a crossover from negative to positive thermal expansion [30]. Our results, as evident from the data presented in Table A1, in general support such conclusions.

**Figure 1:**
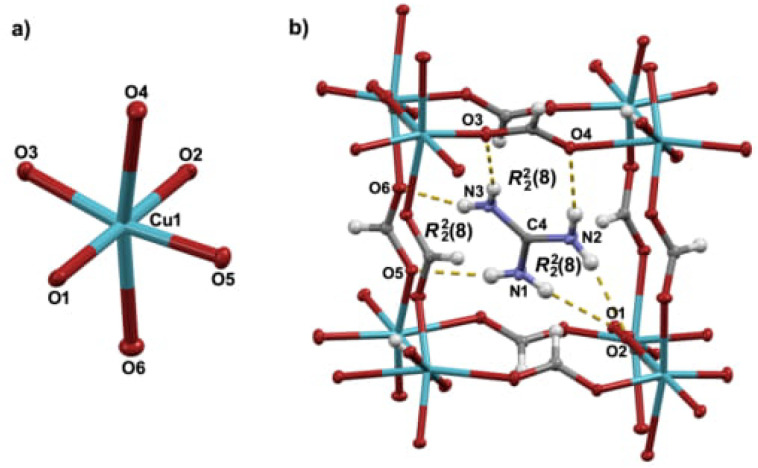
Mercury-ORTEP [34] POV-Ray [35] rendered view of (**a**) Jahn-Teller distorted octahedral environment of Cu^2+^ cation in GuaCuF; (**b**) The guanidinium cation residing in the pseudo-cubic cavity whose size is determined by positions of eight bridged copper (II) ions. Guanidinium cation is anchored in the cavity by six hydrogen bonds. Ellipsoids are drawn at the 50% probability level while H-atoms are shown as spheres of arbitrary size. In (**b**) hydrogen bonds are presented by an array of yellow cylinders.

**Figure 2:**
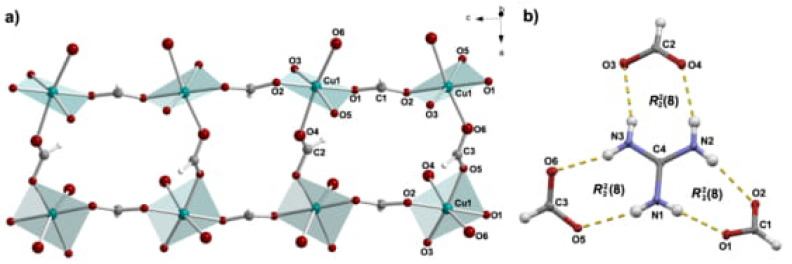
(**a**) The chains of CuO_4_ units (defined by two short and two medium Cu–O bond lengths). The chains, which run along the crystallographic *c*-axis are formed *via* two medium Cu–O bonds of the formate anions. The chains further associate through long and short Cu–O distances, which involve formate anions. Guanidinium cations are not shown for clarity reasons. (**b**) Hydrogen bonds and the related graph-set notations formed by guanidinium cation. Hydrogen bonds are presented by yellow dashed lines.

The structure of the title compound, GuaCuF, consists of the anionic framework [Cu(HCOO)${}_{3}$]^–^ whose pseudo-cubic cavities are populated by guanidinium cations. The Cu^2+^ is found in the Jahn-Teller distorted octahedral environment, resulting in 4 + 2 geometry (Figure 1). The Cu^2+^ cations are mutually connected through formate bridges. A useful description of the structure of GuaCuF, especially given its magnetism, is the one that considers the square-planar CuO_4_ units, defined by the shorter Cu–O bonds, that is, two short and two medium-length bonds. Accordingly, the structure can be perceived as composed of the chains containing CuO_4_ units, which run along the crystallographic *c*-axis (Figure 2a). The Cu^2+^ ions within the chains are connected through *anti-anti* formate bridges, which include medium-length Cu–O bonds, while the intrachain Cu ··· Cu distance is 5.643(3) Å. The neighbouring chains are linked via long and short Cu–O bonds, and the interchain Cu ··· Cu distances are 6.1617(5) Å and 6.1838(5) Å. The main geometrical parameters of GuaCuF structure at 100 K roughly resembles the scenario observed at room temperature [18].

As mentioned previously, guanidinium cations are anchored within the pseudo-cubic framework cavities *via* strong hydrogen bonds (Figure 1b and Figure 2b and Table A3). More precisely, each guanidinium cation participates in six fairly strong N–H···O hydrogen bonds in total (there are three non-equivalent R${}_{2}^{2}\left(8\right)$ rings).

### 3.2. Magnetic Susceptibility

**Figure 3:**
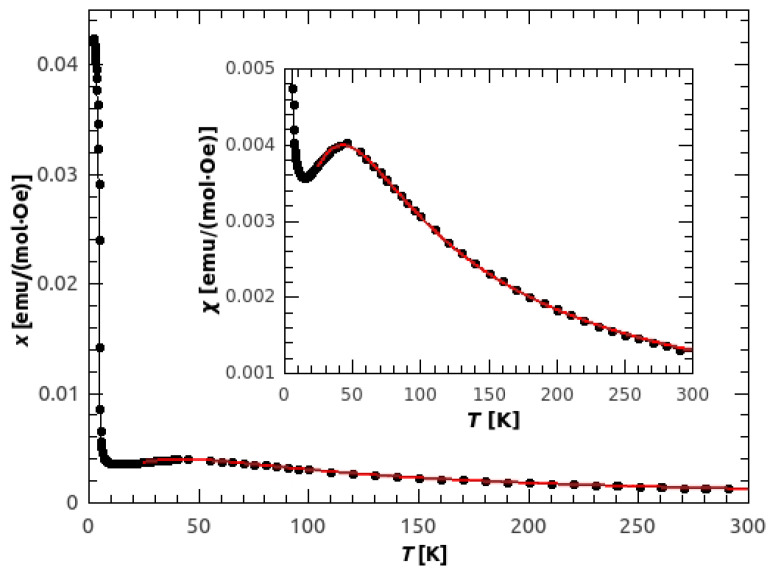
Temperature dependence of molar susceptibility of the powder sample of GuaCuF, measured in 1 kOe. Red line represents the Bonner-Fisher spin-chain fit with mean field correction for the inter-chain interactions.

**Figure 4:**
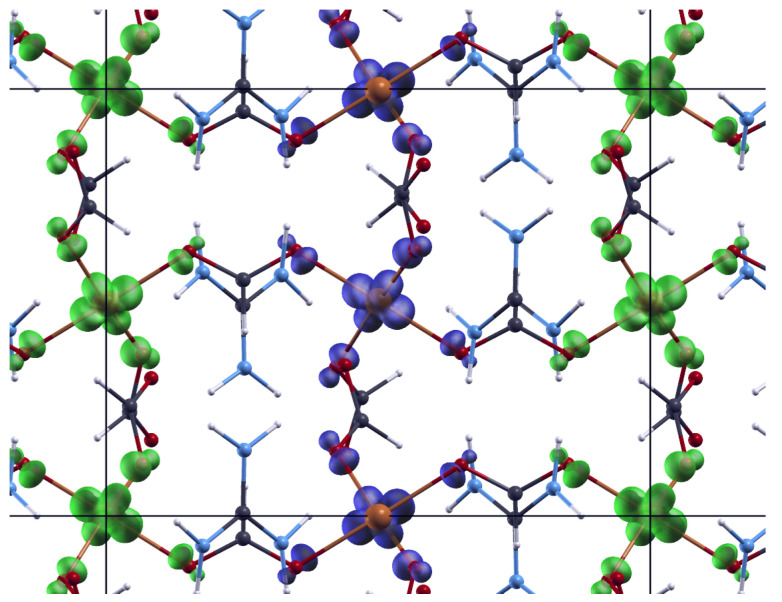
Spin polarization density (difference of electronic density of spin up and spin down) of ground state. Spin up polarization is shown with blue isosurfaces and spin down polarization is shown with green isosurfaces. Horizontal is crystallographic *c* direction and vertical is crystallographic *a*+*b* direction.

Susceptibility obtained from the temperature dependence of magnetization M(T) in 1000 Oe is shown in Figure 3. The broad peak, with the maximum at 45 K, points to the existence of antiferromagnetic (AFM) chains with relatively strong intra-chain coupling. The strength of the AFM interaction was obtained by fitting the data with the Bonner-Fisher formula for antiferromagnetic (AFM) spin 1/2 chains [42]:

(1) $$\chi_{chain}=\frac{N_{A}\beta^{2}g^{2}}{k_{B}T}\frac{0.25+0.074975x+0.075235x^{2}}{1+0.9931x+0.172135x^{2}+0.757825x^{3}},$$

where $x=\left|J\right|/k_{B}T$ and *J* is the super-exchange interaction between the neighbouring copper ions inside the chain, defined by the Hamiltonian $H=-J\sum S_{i}\cdot S_{i+1}$. The values of J=(−65.5±0.1) K and g=(2.18±0.02) were obtained, with the RMSE (Root Mean Squared Error) of $1.37\cdot 10^{-5}$. If we take into account the secondary bonds (super-exchange between the chains) as a mean field correction, the susceptibility becomes:

(2) $$\chi =\frac{\chi_{chain}}{1-\frac{zj}{N_{A}\beta^{2}g^{2}}\chi_{chain}},$$

where *j* is the inter-chain interaction and z=4 is number of the nearest chains. The obtained values are following: J=(−66.5±0.3) K, g=(2.17±0.02) and j=(8.3±2.6) K, with reduced $RMSE=1.23\cdot 10^{-5}$. From these results we can describe the basic magnetic structure as an A-type AFM, where antiferromagnetic chains are mutually weakly coupled by ferromagnetic interaction. This is consistent with the magnetic ground state given by DFT calculations, (Figure 4) where the obtained super-exchange interactions were $J_{c}$ = −886 K, and $J_{ab}$ = 296 K. The reason for such large values is the use of PBE exchange-correlation functional that tends to delocalize *d* electrons and, thus, overestimate super-exchange. However, the ground state is correctly predicted and better agreement would be obtained by using DFT corrected by Hubbard term [43] or DFT functional containing part of exact exchange [44]. Such calculations were performed in Reference [19] with resulting $J_{c}$ = −63.3 K and $J_{ab}$ = 12 K.

The magnetic ground state of an A-type AFM can be explained by looking at the orbitals. The antiferro-orbital ordering within the ab plane and ferro-orbital ordering along the chain, along with the Goodenough-Kanamori-Anderson rules points to ferromagnetic coupling within the ab plane and antiferromagnetic coupling along the chain in *c* direction. The detailed explanation can be found in [19].

The previous work on the GuaCuF powder reported magnetic structure of antiferromagnetic chains with the intra-chain interaction of $J_{intra}$ = −68.1 K and weak ferromagnetism resulting from canted spins at temperatures below $T_{N}$ = 4.6 K [18]. In our sample of GuaCuF, we obtained similar value of the strongest super-exchange constant.

### 3.3. Magnetic Anisotropy

**Figure 5:**
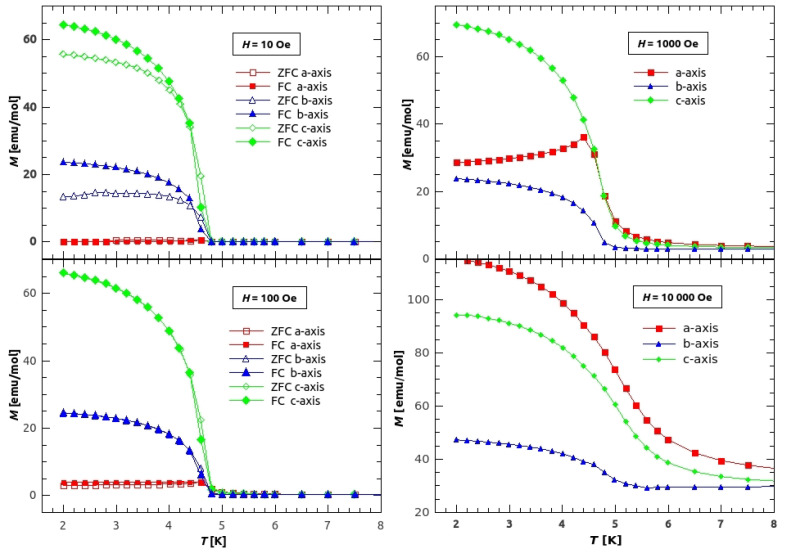
Temperature dependence of magnetization of single crystal GuaCuF in different fields. The magnetization was measured along the applied field. Red rectangles represent magnetization measured along the *a*-axis, blue triangles along the *b*-axis, and green diamonds along the *c*-axis. Empty/filled symbols stand for ZFC/FC curves, respectively.

**Figure 6:**
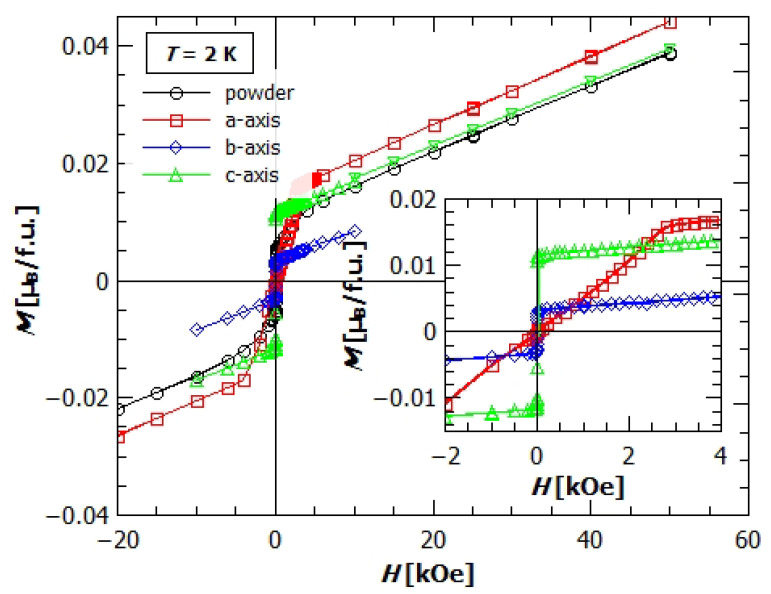
Field dependence of magnetization of the powder and single crystal samples of GuaCuF. Black dots represent powder data, red rectangles the magnetization of single crystal in the direction of the applied field, parallel to *a*-axis, blue diamonds parallel to *b*-axis, and green triangles parallel to *c*-axis.

If we look at the magnetization of the single crystals (Figure 5 and Figure 6), we can see a large difference in magnetization, both in M(T) and in M(H), along different crystallographic axes, which indicates a large magnetic anisotropy of the compound.

On Figure 5, the low temperature magnetization along different crystal axes is shown. On the left side, magnetization measured in 10 and 100 Oe of applied field is shown. From the magnetization in the field of 10 Oe (upper left figure) we have found the critical temperature, the temperature below which the weak ferromagnetic long range order (LRO) is established, to be $T_{N}=4.6$ K (in agreement with the value found in [18]). The irreversibility, by means of difference between the ZFC and FC curves, is noticeable only in the lowest field of 10 Oe for the *b* and *c* direction, while for *a* direction it is still around 25% in the field of 100 Oe. The large difference between the values of magnetization in different directions, below the transition temperature $T_{N}$, indicates large anisotropy with easy axis being in *c* direction and hard axis in *a* direction, concerning at least the macroscopic magnetization. The easy axis in canted weak ferromagnets indicates the direction in which the uncompensated magnetic moment of canted AFM ordered spins is oriented most favourably, so that the same magnetic field applied in directions different from the direction of the easy axis, will produce much lower value of magnetization. However, in this material, higher fields change the direction in which the spins are most favourably canted. On the right side of the Figure 5, the M(T) in fields 1 kOe and 10 kOe are shown. Increasing the field the sample becomes more magnetized in *a* direction. For the field of 1 kOe, it is still easiest to magnetize the sample in *c* direction, but it is easier to cant the spins in *a* direction than in *b* direction. Finally the field of 10 kOe (lower right part of Figure 5) changes the easy axis to *a* direction, while the hard axis stays in *b* direction. It has to be noted, that this discussion covers only the main crystallographic directions due to simplicity, while more detailed research of anisotropy will be continued with other appropriate techniques for magnetic anisotropy.

The field dependence of magnetization of single crystals at 2 K, (Figure 6) clearly shows the anisotropic behaviour discussed above. At the lowest fields, till around 20 Oe, the magnetization in *b* and *c* direction increases rapidly and achieves the values $M_{c}=0.012$ μ${}_{B}$ and $M_{b}=0.003$ μ${}_{B}$ per Cu^2+^ ion. After this initial rapid increase, they continue with slow linear increase with the same slope. The magnetization in *a* direction starts to increase with the field with much smaller rate, achieving only $M_{a}=0.0002$ μ${}_{B}/f.u.$ (one formula unit—f.u. contains one Cu^2+^ ion) at 20 Oe. After 20 Oe it continues with the same slope, and at the field of 800 Oe attains larger value of magnetization than in *b* direction, while at fields larger than 2300 Oe it becomes larger than $M_{c}$. After 3000 Oe, it becomes harder to increase the magnetization further, the change of the magnetization stays linear, and the rate at which it changes with field becomes equal to the slopes of $M_{b}$ and $M_{c}$.

Besides anisotropy, from the M(H) it could also be seen that the saturation is not achieved even in fields of 50 kOe, where the value of magnetisation is only around 0.04 μ${}_{B}/f.u.$ (for S=1/2 of copper, the saturation value should be around 1 μ${}_{B}/f.u.$). This confirms our claim of canted spins and weak ferromagnetism. Higher magnetic fields for studying the possible spin flip/flop processes are unreachable in this setup.

**Figure 7:**
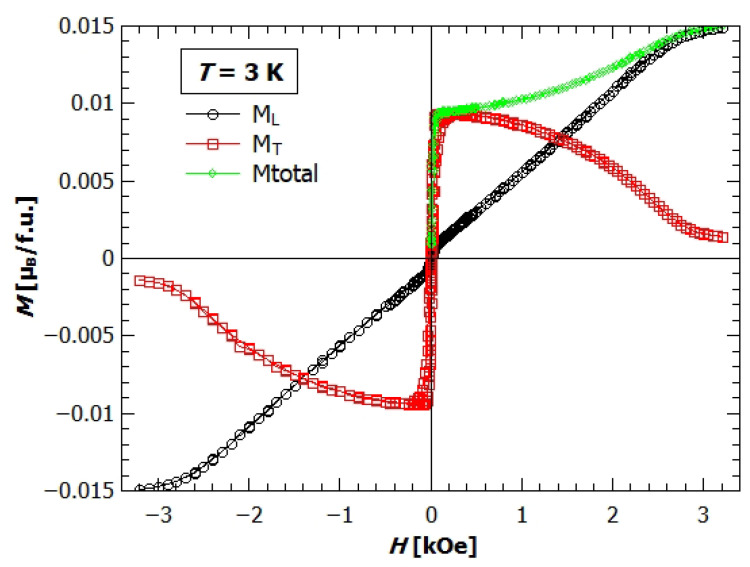
Field dependence of magnetization of single crystal sample of GuaCuF at 3K. Black dots represent the longitudinal magnetization, red rectangles transverse magnetization, and green diamonds total magnetization ($M_{tot}=\sqrt{M_{L}^{2}+M_{T}^{2}}$).

Figure 7 shows the field dependence of magnetization at 3K, measured simultaneously along the field applied in *a* direction (longitudinal magnetization, $M_{L}$) and perpendicular to the field (transverse magnetization, $M_{T}$). With horizontal rotator the crystal is firstly rotated in such a direction, so that the detector coil measures $M_{T}$ in the direction of maximum value of magnetization in the plane perpendicular to the applied field (here that is along *c*-axis, as it is the easy axis). It can be seen that even though the field is applied in *a* direction, the magnetization in the bc plane is larger than in *a* direction. Only the fields higher than 3 kOe manage to overcome the anisotropy of the crystal and magnetize the sample in the direction of vertically applied field, that is, the longitudinal direction. If we look at the total magnetization as a vector, we can calculate its value and angle of direction and observe how do they change with the applied field. The value of total magnetization is shown on the Figure 7 with the green diamonds. For the small fields the vector of total magnetization lies in *c* direction and $M_{tot}$ is almost equal to the $M_{T}$. Increasing the field the direction changes almost linearly towards the *a* direction till around 2.5 kOe, where it is almost parallel to the *a* axis.

Possible reason for anisotropy can be found in the crystal structure. The elongated octahedra, which are alternately canted one from another, influence the local anisotropy around Cu^2+^ ions. More detailed analysis will be made using the techniques more appropriate for the investigation of magnetic anisotropy, as well as for microscopic origins of it, successfully contributing in oxides having similar magnetic complexity [45,46].

### 3.4. Magneto-Electric Study

**Figure 8:**
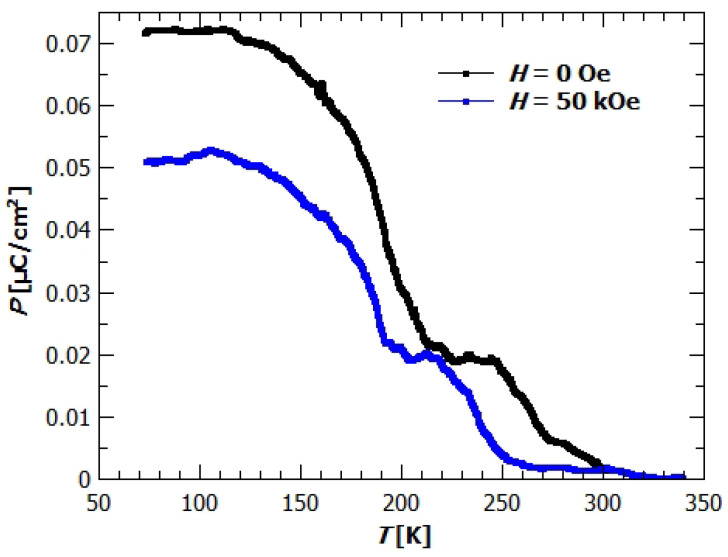
Polarisation of GuaCuF. Measured in c direction while cooling. The black circles represent measurement in zero magnetic field and blue rectangles in 50 kOe magnetic field.

Polarization of the GuaCuF crystal measured in the fields of 0 and 50 kOe is shown on Figure 8. The black dots show spontaneous polarization in zero magnetic field, while the blue rectangles show spontaneous polarization measured in the magnetic field of 50 kOe. The polarization is measured and magnetic field applied in *c* direction. Even though we applied no electric field *E*, just as a consequence of cooling the sample, measurable current was generated at the electrical contacts made on the ab planes of the crystal and integrated with time it gave the polarization of the sample. Because the measurement was done in *E* = 0 while cooling, the gradual formation of the ferroelectric order toward the maximal polarization of the sample is observed. From the Figure 8 can be seen that the value of polarization depends on the value of applied magnetic field. The polarization in 0 Oe is 0.072 μC/cm${}^{2}$, while the field of 50 kOe suppresses the polarization to the value of 0.053 μC/cm${}^{2}$. The ordering of dipole moments happen in two main parts. As we cool down the sample, the polarization starts to rise at around 270 K, and rises to the value of 0.019 μC/cm${}^{2}$ at 245 K. After this initial rise, the ordering of the dipoles abates and the polarization stays almost constant till 210 K, where again the rapid increase of polarization happens. The final value of polarization is achieved at 110 K and amounts 0.072 μC/cm${}^{2}$. Similar behaviour is also seen from the measurements in applied magnetic field. Magnetic field suppresses the ordering of electric dipoles, so that the transitions happen at somewhat lower temperatures and the final value is around 75% of the value in zero magnetic field. Similar effect was also observed in [20], where the polarization was measured in magnetic fields 0, 70 and 110 kOe, applied in ab-plane. There the pyroelectric current was measured with poled samples, and the obtained values of polarization were 0.11 μC/cm${}^{2}$, 0.023 μC/cm${}^{2}$ and 0.011 μC/cm${}^{2}$, respectively. Onset of the ferroelectric ordering was found below the transition temperature $T_{C}=277K$, where a sharp change on P(T) curve was observed. The described difference in behaviour of polarization with temperature and the higher value at which the polarization starts to develop can be understood if we notice the fact that the samples were poled [20]. The electric field enabled easier ordering of the dipoles and thereby full ordering at the higher temperatures. In our case without the influence of applied electric field, spontaneous transition to the ordered polar state was measured, being therefore complementary to the already published results in [20]. Furthermore, these measurements show that the magnetic field can influence polarization even in the paramagnetic state (paramagnetoelectric effect—PME effect). It was suggested that the PME effect appears due to the nonlinear ME coupling via magnetostriction and ferroleastic effects [20].

**Figure A3:**
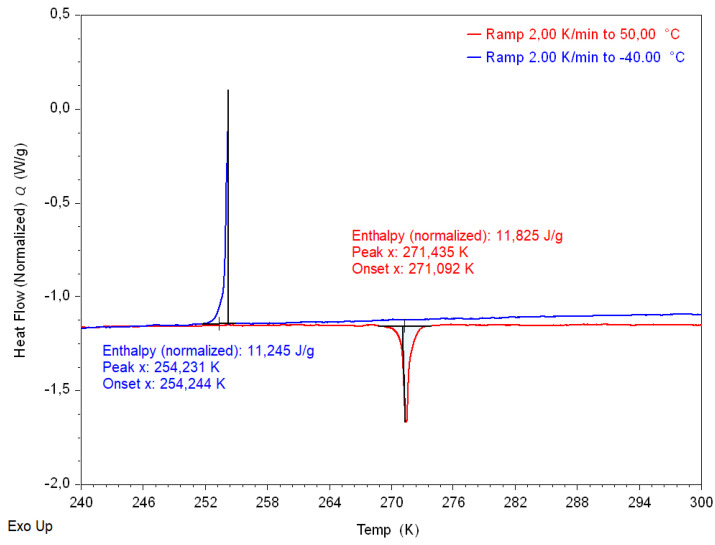
DSC measurement of [C(NH${}_{2}$)${}_{3}$]Cu(HCOO)${}_{3}$ (red—heating (2 K/min); blue—cooling (2 K/min)).

The DSC measurement in the temperature range from 233 K to 323 K unveiled a reversible thermal event (heating onset temperature 271 K) which is in accordance with previously published results [20] and can be assigned to the ferroelectric-paraelectric phase transition (Figure A3).

From the previous theoretical considerations of the mechanism of polarization it was concluded that the main contribution to the polarization comes from the dipole moments induced by the displacements of NH_2_ groups of Gua cation, which couple through the hydrogen bonds with Jahn-Teller distortions and enable the formation of ferroelectric order. More details can be found in [19,20].

From the DFT calculations, the polarization of 0.19 μC/cm${}^{2}$ was obtained (previous reports gave the value od 0.37 μC/cm${}^{2}$ due to different computational setup [19]). To compare it with the experimental value, we have to take into account the twinned nature of our crystal. The size-ratio of two twin components being around 40:60, means that only around 20% of total polarization will not cancel out. The experimental value, *P* = 0.072 μC/cm${}^{2}$ is around 19% of the value obtained in [19], and 38% of the here calculated 0.19 μC/cm${}^{2}$, showing relatively good agreement with the amount of twinned domains whose contributions do not cancel out.

**Figure 9:**
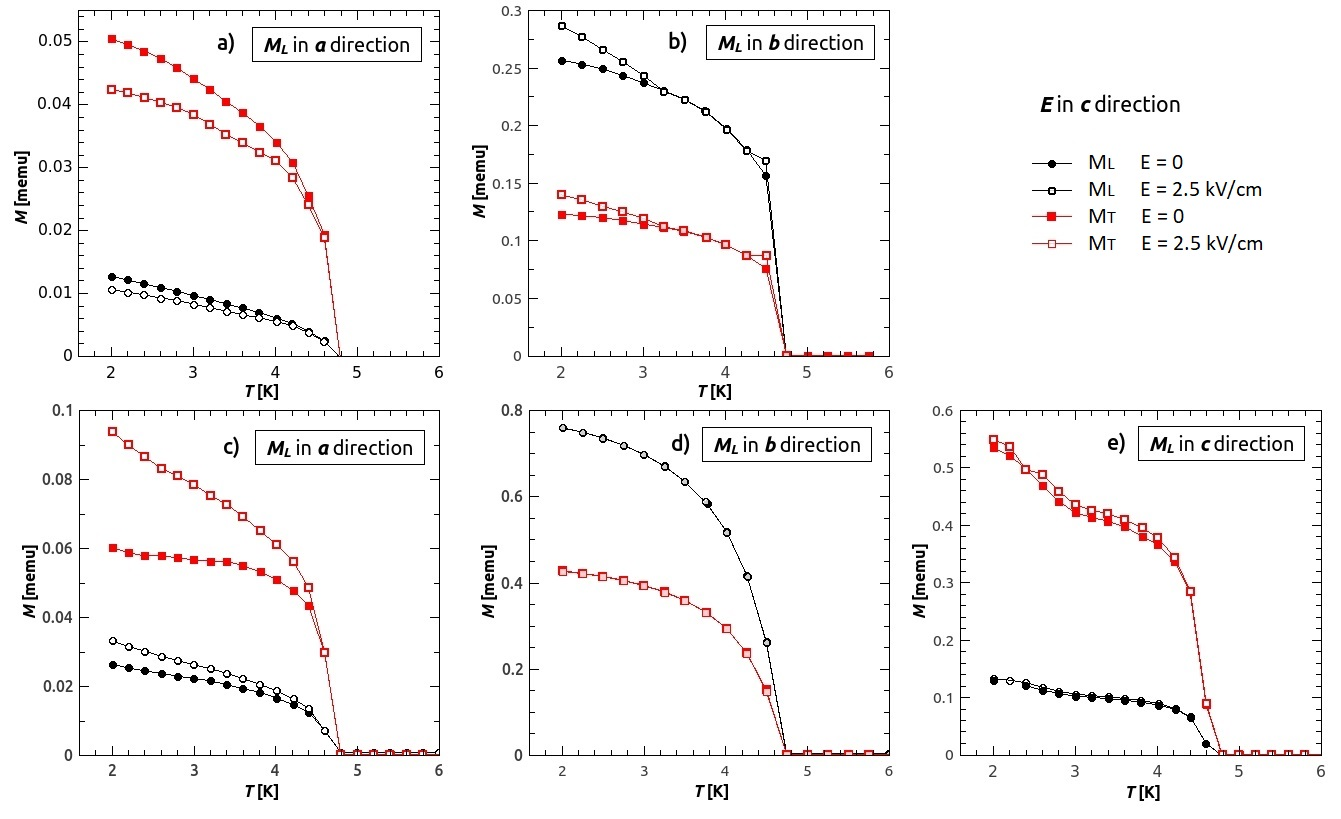
Magnetoelectric effect. Five different measurement setups, (**a**,**b**) measurements in H = 0 Oe, directly after cooling the superconducting magnet (no remanent fields). (**c**–**e**) after using the magnet (unknown remanent field). Black circles represents longitudinal magnetization, while red rectangles transverse magnetization. Filled symbols represent the magnetization measured in zero electric field, while empty symbols magnetization measured in electric field (E≈ 2.5 kV/cm) applied in *c* direction.

The influence of the electric field *E* on magnetization was observed as a change in the value of magnetization *M* at the temperatures below $T_{N}$. On Figure 9, measurements of magnetization with (empty symbols) and without (filled symbols) the electric field applied in *c* direction is shown. Five figures represent five different measurement setups where the magnetization was measured along *a* (Figure 9a,c), *b* (Figure 9b,d) and *c* crystal axis (Figure 9e). Measurements were done in different remanent fields and different attempts of orienting the crystal. The measurements on Figure 9a,b were done when the superconducting magnet was first time cooled, before turning on the magnet so that there was no remanent magnetic field present, while Figure 9c–e measurements were done after the magnet was used, thereby having some remanent field which was not possible to quantify or remove completely. For each setup, the transverse component of magnetization (the maximum value of magnetization in the plane perpendicular to the magnetic field direction) was measured at the same time as the longitudinal component. Black circles represent longitudinal, while red rectangles transverse component of magnetization. Obviously, the vector of magnetization is changed solely by electric field, since we did not apply any magnetic field. For measurements on Figure 9a,b the magnetometer’s superconducting magnet in a fresh state is used having no remanent magnetic field and the crystal was magnetized only by the Earths magnetic field, while for measurements on Figure 9c–e there is some unknown remanent field. This still makes complications in interpretation since crystal is oriented differently with respect to the Earths magnetic field and remanent field, making it hard to compare the measurements and deduce precise conclusions about the exact change of the vector of magnetization. In that sense our experiment is complementary to the findings in [20] where they have applied relatively large magnetic field (1 kOe) during magnetoelectric measurement and obtained relative change of magnetization of 7%. Our experiment is different and has different results, since we changed the almost spontaneous magnetic state using the electric field alone.

The biggest magneto-electric (ME) effect was observed when the magnetization was measured in *a* direction (Figure 9a,c), with the relative change in magnetization of 17% and 21%, respectively. Slightly lower relative change, 16%, was observed in component transverse to *a* direction on the Figure 9a, while on the Figure 9c, the relative change in transverse direction was 36%. The measurements in *b* direction showed the relative change of 10% in longitudinal and 12% in transverse component of magnetization for the setup in Figure 9b, while there was no change of magnetization neither in longitudinal nor in transverse direction for the measurement shown on Figure 9d. The electric field had no influence on magnetization when it was measured in *c* direction. From the observed behaviour we could conclude that the electric field has an influence on the magnetization mostly in *a* direction (or the direction near *a* direction), and all the observed changes in other directions happened because of the non perfect orientation of the crystal on the sample holder (it was easiest to orient the crystal in *c* direction, therefore we measured only $M_{c}$ in longitudinal direction and $M_{b}$ in transverse direction—*a* being the hard axis). If the sample was not positioned ideally, meaning that there was a small component of $M_{a}$ in transverse direction, the change in $M_{T}$ could be explained. It is possible that the remanent field hinders the effect that electric field has on magnetization in the longitudinal direction, therefore enabling the change in $M_{T}$ to be larger than in $M_{L}$ (no remanent field in transverse direction), (Figure 9c). Better orientation of the crystal in the setups on Figure 9d,e and higher remanent field caused no visible ME effect in *b* and *c* directions. Electric field applied in the other main crystallographic directions showed no measurable ME effect. Both longitudinal and transverse component of magnetization change with the application of the electric field, therefore we conclude that the electric field changes the vector of magnetization.

Additionally, it is important to remark that the change in magnetization which occurred from applying the electric field, was not returned to its original value with turning off the field or even with reversing the field. Only by heating the sample above the ferroelectric transition and then cooling it in zero electric field, were we able to obtain the initial magnetization, the same one as prior to application of the electric field. Origin of the magneto-electric effect could be found in the detailed explanations of electric polarization mechanism [19]. Electric field or electric ordering induced rotations of guanidinium ions produce some slight distortions of formate bridges, that changes the amounts of the symmetric and anti-symmetric super-exchange interactions as well as eventual local anisotropy around Cu. All of this can change the vector of magnetization coming from canted magnetic moments, and provide the reasons for magneto-electric effect. This mechanism is still not know completely and motivates for further research of the microscopic origins of this relatively strong magneto-electric effect.

## 4. Conclusions

In this work, we investigate a single crystal of guanidinium copper (II) formate [C(NH${}_{2}$)${}_{3}$]Cu(HCOO)${}_{3}$ and give some novel insights concerning the structural, magnetic, electric and magneto-electric properties of this interesting magnetoelectric multiferroic material.

Magnetic susceptibility points to the existence of antiferromagnetic spin-chains of Cu (II) and their much weaker ferromagnetic interaction with neighbouring chains. The performed ab-initio colinear DFT calculation confirms in first approximation the magnetic ground state determined with super-exchange interactions over the formate bridges and explains the ferromagnetic coupling of the antiferromagnetic spin-chains running along the *c* direction into an overall antiferromagnetic-like ordered lattice below 4.6 K.

More thorough analysis of magnetization measured on a single crystal shows that system in ground state is actually a canted antiferromagnet and that magnetization coming from the canted magnetic moments has a very large anisotropy, with easy axis in *c* direction and *a* as a hard direction (small fields produce almost no magnetic moment along *a*). Special benefits come from the measurements using the transverse moment, besides the longitudinal which is standard in the magnetometers. Origins of canted magnetic moments and anisotropy of magnetization could be found in the canting of the coordination octahedra around copper (II) ions throughout the crystal lattice.

With cooling down in zero electric field the generation of spontaneous polarization is found below 270 K and 210 K even in this twinned crystal, and the effect of magnetic field on its value is observed also in the paramagnetic phase. Polarization is measured in *c* direction since DFT calculations predicted this vector. Measured value is somewhat smaller than the DFT value and smaller than it should be in a non-twinned crystal.

The considerable magnetoelectric coupling below the magnetic transition temperature was measured with different directions of electric field and orientations of the crystal in magnetic field, giving altogether the new light onto the magnetoelectric effect in this material. Although the polar and magnetic order establish at very different temperatures, coupling between these two orders is considerably high.

Along with these novel results about magnetic anisotropy and magnetoelectric effect in guanidinium copper (II) formate, their further research is needed to fully understand the microscopic origins of these interesting phenomena in this hybrid metal-organic magnetoelectric multiferroic.

## Appendix A. Structural Features

From the X-ray diffraction analysis (Figure A1), many different parameters can be extracted. Selected crystallographic and refinement data is given in Table A1. For discussions of the physical properties and comparison to other known systems, a bond lengths and bond angles are useful, therefore their values are given in Table A2 for selected bonds. Also, the hydrogen bonds play an important role in forming and stabilization of the crystal lattice, therefore their geometry is described with parameters given in Table A3.

**Table A1:** Crystallographic data for compound **1**.

Complex	1
Chemical formula	C_4_H_9_CuN_3_O_6_
*M*_r_	258.69
Crystal system, colour and habit	orthorhombic, blue, prism
Crystal dimensions/mm^3^	0.06 × 0.08 × 0.21
Space group	*Pna*2_1_(No. 33)
*Z*	4
Unit cell parameters:	
*a* /Å	8.4350(2)
*b* /Å	9.0145(2)
*c* /Å	11.2820(4)
α/°	90
β/°	90
γ/°	90
*V*/Å^3^	857.85(4)
*D*_calc_/g cm^–3^	2.003
μ/mm^–1^	2.558
*F*(000)	524
No. refined parameters, *N*_p_/restraints	146/6
Reflections collected, unique (*R*_int_), observed [*I* ≥ 2σ(*I*)]	8136, 2072 (0.016), 1996
*R*_1_^a^ [*I* ≥ 2σ(*I*)]	0.0202
*g*_1_, *g*_2_ in *w* ^b^	0.0326, 0.2077
*wR*_2_^c^ (all data)	0.0554
Goodness of fit on *F*^2^, *S* ^d^	1.08

^a^*R* = Σ||*F*_o_| – |*F*_c_||/ Σ|*F*_o_|; ^b^ *w* = 1/[σ^2^(*F*_o_^2^) + (*g*_1_*P*)^2^ + *g*_2_*P*] where *P* = (*F*_o_^2^ + 2*F*_c_^2^)/3; ^c^ *wR* = {Σ[*w*(*F*_o_^2^ − *F*_c_^2^)^2^]/Σ[*w*(*F*_o_^2^)^2^]}^1/2^; ^d^ *S =* {Σ[*w*(*F*_o_^2^ – *F*_c_^2^)^2^]/(*N*_r_ − *N*_p_)}^1/2^ where *N*_r_ = number of independent reflections, *N*_p_ = number of refined parameters.

**Table A2:** Selected bond lengths and angles for [C(NH_2_)_3_][Cu(HCOO)_3_].

Bond lengths			
A−B	*d*(A−B)/Å	A−B	*d*(A−B)/Å
Cu1–O1	1.992(3)	O5–C3 ^1^	1.272(3)
Cu1–O2	1.991(3)	C2–O3 ^2^	1.269(3)
Cu1–O3	1.953(2)	O1–C1 ^3^	1.254(6)
Cu1–O4	2.3597(19)	O4–C2	1.238(3)
Cu1–O5	1.9674(19)	O2–C1	1.251(6)
Cu1–O6	2.331(2)	O6–C3	1.235(3)
		N3–C4	1.321(2)
		N2–C4	1.306(12)
		C4–N1	1.352(13)
Bond angles			
A–B–C	*∠*(A–B–C)/°		
O1– Cu1–O2	179.36(8)	C3–O6–Cu1	134.59(19)
O1– Cu1–O3	89.54(9)	C1–O2–Cu1	121.3(2)
O1– Cu1–O4	88.91(8)	C2–O4–Cu1	130.41(18)
O1– Cu1–O5	89.57(12)	C3 ^1^–O5–Cu1	128.22(18)
O1– Cu1–O6	90.70(12)	O4–C2–O3 ^2^	124.1(3)
O2– Cu1–O3	89.95(13)	C1 ^3^–O1–Cu1	121.6(2)
O2–Cu1–O4	91.44(11)	N2–C4–N3	121.7(10)
O2–Cu1–O5	91.02(9)	N2–C4–N1	119.87(19)
O2–Cu1–O6	89.07(8)	N3–C4–N1	118.3(11)
O3–Cu1–O4	84.94(8)	C2 ^4^–O3–Cu1	129.3(2)
O3–Cu1–O5	166.90(8)	O2–C1–O1 ^5^	124.0(2)
O3–Cu1–O6	106.03(6)	O6–C3–O5 ^6^	124.0(2)
O4–Cu1–O5	81.97(6)		
O4–Cu1–O6	169.02(8)		
O5–Cu1–O6	87.05(9)		

Symmetry codes: ^1^ −1/2 + *x*,1/2−*y*, + *z*; ^2^ −1/2 + *x*,3/2−*y*, + *z*; ^3^ 1−*x*,1−*y*,−1/2 + *z*; ^4^ 1/2 + *x*,3/2−*y*, + *z*; ^5^ 1−*x*,1−*y*,1/2 + *z*; ^6^ 1/2 + *x*,1/2−*y*, + *z*.

**Table A3:** The geometry of hydrogen bonds (Å, ^o^) for [C(NH_2_)_3_][Cu(HCOO)_3_].

D–H⋯A	D–H	H⋯A	D⋯A	*∠*D–H⋯A	Symmetry Code
N1–H1A⋯O5	0.84(3)	2.15(3)	2.969(3)	167(3)	−x,1−y,1/2 + z
N1–H1B⋯O1	0.820(19)	2.12(2)	2.925(3)	170(3)	1/2−x,−1/2 + y,1/2 + z
N2–H2A⋯O4	0.87(3)	2.07(3)	2.920(3)	165(3)	-
N2– H2B⋯O2	0.818(18)	2.18(2)	2.981(3)	167(3)	−1/2 + x,1/2−y,z
N3–H3A⋯O6	0.84(3)	2.10(3)	2.911(4)	165(3)	1/2−x,1/2 + y,1/2 + z
N3–H3B⋯O3	0.83(3)	2.17(3)	2.970(4)	164(3)	−1/2 + x,3/2−y,z

## Appendix B. Electric Properties

Temperature dependence of the pyroelectric current of GuaCuF measured while cooling in 0 and 50 kOe magnetic field is shown on Figure A2. For configuration of electric contacts on the crystal see Section 2.5. No electric field was used, neither during measurements, nor for the poling purposes.

## Appendix C. Thermodynamic Properties

Differential Scanning Calorimetry (DSC) measurement on a single crystal of GuaCuF is shown on Figure A3. Observed reversible thermal event was associated with a ferroelectric-paraelectric phase transition.

## References

[1] Fiebig M. (2005). Revival of the magnetoelectric effect. J. Phys. D Appl. Phys..

[2] Khomskii D. (2009). Classifying multiferroics: Mechanisms and effects. Physics.

[3] Dong S., Liu J.M., Cheong S.W., Ren Z. (2015). Multiferroic materials and magnetoelectric physics: Symmetry, entanglement, excitation, and topology. Adv. Phys..

[4] Wang K., Liu J.-M., Ren Z. (2009). Multiferroicity: The coupling between magnetic and polarization orders. Adv. Phys..

[5] Gajek M., Bibes M., Fusil S., Bouzehouane K., Fontcuberta J., Barthélémy A., Fert A. (2007). Tunnel junctions with multiferroic barriers. Nat. Mater..

[6] Ramesh R., Spaldin N.A. (2007). Multiferroics: Progress and prospects in thin films. Nat. Mater..

[7] Wei Y., Gao C., Chen Z., Xi S., Shao W., Zhang P., Chen G., Li J. (2016). Four-state memory based on a giant and non-volatile converse magnetoelectric effect in FeAl/PIN-PMN-PT structure. Sci. Rep..

[8] Hill N.A. (2000). Why are there so few magnetic ferroelectrics?. J. Phys. Chem. B.

[9] Hill N.A., Filippetti A. (2002). Why are there any magnetic ferroelectrics?. J. Magn. Magn. Mater..

[10] Catalan G., Scott J.F. (2009). Physics and Applications of Bismuth Ferrite. Adv. Mater..

[11] Rogez G., Viart N., Drillon M. (2010). Multiferroic Materials: The Attractive Approach of Metal–Organic Frameworks (MOFs). Angew. Chem. Int. Ed..

[12] Li W., Wang Z., Deschler F., Gao S., Friend R.H., Cheetham A.K. (2017). Chemically diverse and multifunctional hybrid organic–inorganic perovskites. Nat. Rev. Mater..

[13] Cheetham A.K., Rao C.N.R. (2007). There’s room in the middle. Science.

[14] Li W., Stropa A., Wang Z.M., Gao S. (2020). Hybrid Organic-Inorganic Perovskites.

[15] Wang Z., Zhang B., Otsuka T., Inoue K., Kobayashi H., Kurmoo M. (2004). Anionic NaCl-type frameworks of Mn^II^(HCOO)_3_^−^, templated by alkylammonium, exhibit weak ferromagnetism. Dalton Trans..

[16] Jain P., Dalal N.S., Toby B.H., Kroto H.W., Cheetham A.K. (2008). Order-Disorder Antiferroelectric Phase Transition in a Hybrid Inorganic-Organic Framework with the Perovskite Architecture. J. Am. Chem. Soc..

[17] Sánchez-Andújar M., Presedo S., Yáñez-Vilar S., Castro-García S., Shamir J., Señarís-Rodríguez M.A. (2010). Characterization of the order-disorder dielectric transition in the hybrid organic-inorganic perovskite-like formate Mn(HCOO)_3_[(CH_3_)_2_NH_2_]. Inorg. Chem..

[18] Hu K.-L., Kurmoo M., Wang Z., Gao S. (2009). Metal–Organic Perovskites: Synthesis, Structures, and Magnetic Properties of [C(NH_2_)_3_][M^II^(HCOO)_3_] (M=Mn, Fe, Co, Ni, Cu, and Zn; C(NH_2_)_3_=Guanidinium). Chem. Eur. J..

[19] Stroppa A., Jain P., Barone P., Marsman M., Perez-Mato J.M., Cheetham A.K., Kroto H.W., Picozzi S. (2011). Electric Control of Magnetization and Interplay between Orbital Ordering and Ferroelectricity in a Multiferroic Metal–Organic Framework. Angew. Chem. Int. Ed..

[20] Tian Y., Stroppa A., Chai Y.-S., Barone P., Perez-Mato M., Picozzi S., Sun Y. (2015). High-temperature ferroelectricity and strong magnetoelectric effects in a hybrid organic–inorganic perovskite framework. Phys. Status Solidi RRL.

[21] CrysAlisPro 1.171.40.67a (Rigaku OD, 2019), CrysAlis PRO, Agilent Technologies, Version 1.171.37.35 (release 13-08-2014 CrysAlis171 .NET) (compiled Aug 13 2014, 18:06:01). https://www.rigakuxrayforum.com/.

[22] Clark R.C., Reid J.S. (1995). The analytical calculation of absorption in multifaceted crystals. Acta Cryst..

[23] Sheldrick G.M. (2008). A short history of SHELX. Acta Cryst..

[24] Sheldrick G.M. (2015). Crystal structure refinement with SHELXL. Acta Cryst..

[25] Farrugia L.J. (2012). WinGX and ORTEP for Windows: An update. J. Appl. Cryst..

[26] Gui D., Ji L., Muhammad A., Li W., Cai W., Li Y., Li X., Wu X., Lu P. (2018). Jahn–Teller Effect on Framework Flexibility of Hybrid Organic–Inorganic Perovskites. J. Phys. Chem. Lett..

[27] Viswanathan M. (2019). Insights on the Jahn–Teller distortion, hydrogen bonding and local environment correlations in a promised multiferroic hybrid perovskite. J. Phys. Condens. Matter.

[28] Viswanathan M. (2018). Enhancement of the guest orderliness in a low-symmetric perovskite-type metal–organic framework influenced by Jahn–Teller distortion. Phys. Chem. Chem. Phys..

[29] Viswanathan M. (2018). Disorder in the hydrogen-atoms uninvolved in hydrogen bonds in a metal–organic framework. Phys. Chem. Chem. Phys..

[30] Viswanathan M. (2018). Neutron diffraction studies on the thermal expansion and anomalous mechanics in the perovskite-type [C(N_D2_)_3_]Me^2+^(DCOO)_3_[Me = Cu, Mn, Co]. Phys. Chem. Chem. Phys..

[31] Viswanathan M. (2019). Stability of Hydrogen Bonds in the Metal Guanidinium Formate Hybrid Perovskites: A Single-Crystal Neutron Diffraction Study. Cryst. Growth Des..

[32] Spek A.L. (2003). Single-crystal structure validation with the program PLATON. J. Appl. Cryst..

[33] Spek A.L. (2009). Structure validation in chemical crystallography. Acta Cryst..

[34] Macrae C.F., Bruno I.J., Chisholm J.A., Edgington P.R., McCabe P., Pidcock E., Rodriguez-Monge L., Taylor R., Towler M., Van der Streek J. (2008). Mercury CSD 2.0—New features for the visualization and investigation of crystal structures. J. Appl. Crystallogr..

[35] (2021). Persistence of Vision (TM) Raytracer.

[36] Diamond—Crystal and Molecular Structure Visualization, Crystal Impact—Dr. H. Putz and Dr. K. Brandenburg GbR, Kreuzherrenstr. 102, 53227 Bonn, Germany. http://www.crystalimpact.com/diamond.

[37] Giannozzi P., Baroni S., Bonini N., Calandra M., Car R., Cavazzoni C., Ceresoli D., Chiarotti G.L., Cococcioni M., Dabo I. (2009). QUANTUM ESPRESSO: A modular and open-source software project for quantum simulations of materials. J. Phys. Condens. Matter.

[38] Giannozzi P., Andreussi O., Brumme T., Bunau O., Buongiorno Nardelli M., Calandra M., Car R., Cavazzoni C., Ceresoli D., Cococcioni M. (2017). Advanced capabilities for materials modelling with Quantum ESPRESSO. J. Phys. Condens. Matter.

[39] Garrity K.F., Bennett J.W., Rabe K.M., Vanderbilt D. (2014). Pseudopotentials for high-throughput DFT calculations. Comput. Mater. Sci..

[40] Perdew J.P., Burke K., Ernzerhof M. (1996). Generalized Gradient Approximation Made Simple. Phys. Rev. Lett..

[41] King-Smith R.D., Vanderbilt D. (1993). Theory of polarization of crystalline solids. Phys. Rev. B.

[42] Khan O. (1993). Molecular Magnetism.

[43] Kanižaj L., Molčanov K., Torić F., Pajić D., Lončarić I., Šantić A., Jurić M. (2019). Ladder-like [CrCu] coordination polymers containing unique bridging modes of [Cr(C_2_O_4_)_3_]${}_{3}^{3-}$ and [Cr_2_O_7_]^2−^. Dalton Trans..

[44] Jurić M., Androš Dubraja L., Pajić D., Torić F., Zorko A., Ozarowski A., Despoja V., Lafargue-Dit-Hauret W., Rocquefelte X. (2017). Experimental and Theoretical Investigation of the Anti-Ferromagnetic Coupling of Cr(III) Ions through Diamagnetic -O-Nb(V)-O- Bridges. Inorg. Chem..

[45] Herak M., Zorko A., Pregelj M., Zaharko O., Posnjak G., Jagličić Z., Potočnik A., Luetkens H., van Tol J., Ozarowski A. (2013). Magnetic order and low-energy excitations in the quasi-one-dimensional antiferromagnet CuSe_2_O_5_ with staggered fields. Phys. Rev. B.

[46] Živković I., Djokić D.M., Herak M., Pajić D., Prša K., Pattison P., Dominko D., Micković Z., Cinčić D., Forró L. (2012). Site-selective quantum correlations revealed by magnetic anisotropy in the tetramer system SeCuO_3_. Phys. Rev. B.

